# Genetic dissection of QTL for important agronomic traits and fine-mapping of qGL4 and qGW6 based on a short-width grain rice CSSL-Z691

**DOI:** 10.3389/fpls.2025.1539625

**Published:** 2025-03-10

**Authors:** Zhaopeng Yu, Guangyi Xu, Keying Xie, Zhuang Xie, Dachuan Wang, Linlu Tan, Yinghua Ling, Guanghua He, Fangming Zhao

**Affiliations:** Rice Research Institute, Academy of Agricultural Science, Southwest University, Chongqing, China

**Keywords:** rice, chromosome segment substitution lines, single-segment substitution lines, QTL mapping, fine-mapping, grain size

## Abstract

Rice chromosome segment substitution lines (CSSLs) are ideal for creating natural variation and dissecting complex quantitative traits. In addition, it builds a bridge for molecular breeding and accurate identification of quantitative trait loci (QTLs). In this study, to construct an *indica* rice library of single-segment substitution lines (SSSLs) spanning the whole genome, a rice CSSL-Z691 carrying four substitution segments (4.07 Mb of average length) was identified by marker-assisted selection (MAS) from *indica* restorer line “Jinhui35” in the “Xihui18” genetic background. Compared with large panicle type Xihui18, seed setting ratio, grain width, and 1000-grain weight increased in Z691. In contrast, the number of primary branches, spikelet number per panicle, grain number per panicle, grain length, rate of length to width, and yield per plant decreased in Z691. Then, 11 QTLs were identified in the secondary F_2_ population from Xihui18/Z691. Again, four QTLs (*qGW6*, *qGL4*, *qRLW4*, and *qGWT4*) were validated by three SSSLs (S1–S3) developed in F_3_. In addition, 11 new QTLs were detected by the three SSSLs that were not identified in the F_2_ population. Moreover, the different QTLs in D1–D3 showed various genetic models. Some QTLs, e.g., *qGWT6* (*a* = 0.96 g) and *qGWT7* (*a* = −0.29 g), displayed independent inheritance, while others exhibited various epistatic interactions. Thus, it is vital to identify different QTLs and their genetic models. Resolving the epistasis effects among different QTLs is crucial for screening QTLs for breeding by design. Finally, *qGL4* and *qGW6* were fine-mapped to 160- and 240-kb intervals on chromosomes 4 and 6, and two candidate genes were determined by DNA sequencing. These results provide valuable genetic and breeding materials for cloning *qGL4* and *qGW6* and for future molecular breeding by design.

## 1 Introduction

Rice (*Oryza sativa* L.) is the staple food for more than one-half of the world’s population (Yi and Li, 2022). High yield and high quality are the most essential elements in rice breeding from ancient times until now (Zhang et al., 2024a; You et al., 2022). The formation mechanism of rice yield is complex (Zuo and Li, 2014). The effective number of panicles, grain number per panicle, and grain weight mainly control rice yield (Li et al., 2021). At the same time, plant height, plant type, panicle length, grain size, and other factors are also closely related to yield (Wang et al., 2012). However, these agronomic traits are often controlled by multiple genes and influenced by the environment (Abe et al., 2012). In the post-genomic era, the concept of “breeding by design”, which aims to utilize favorable alleles in the crop genome to design and breed varieties for people’s needs, has been proposed. This concept’s core utilizes natural variation (Zhang, 2021). Notably, “breeding by design” combines theoretical research on quantitative trait locus (QTL) mapping with breeding programs, which is essential for rice molecular breeding and genetic improvement (Xu et al., 2021).

As rice chromosome segment substitution lines (CSSLs) differ from the recipient parent in only one or a few substitution segments identified by known molecular markers, they serve as a valuable resource for gene dissection and superior gene pyramiding (Zong et al., 2012). CSSLs enable the precise improvement of parental lines (maintenance and restoration lines) in hybrid rice to facilitate the strategic and efficient development of superior hybrid rice varieties tailored to specific breeding requirements. However, the first step to realizing this great strategy is to dissect and clone the genes for related traits and then study their functions and molecular mechanisms. Traits associated with rice yield are controlled by genes and environment, with genes playing a more significant role in regulation (Zhao et al., 2022). Much research has been conducted on many of the QTLs that determine rice yield by previous generations. For example, *GL3.1* reduces the expression of *Cyclin-T1* protein in rice through a certain regulatory pathway to shorten the length of rice grains (Fan et al., 2006). *GS5* can make endocytosis difficult in rice by preventing the interaction of two related genes (Xu et al., 2015). Reduced or no expression of *OsTB1* increased rice tiller number (Takeda et al., 2003). *OsMPK6* is essential in regulating rice grain size by promoting cell proliferation in spikelet hulls (Bai et al., 2024; Liu et al., 2015; Xu et al., 2018). Natural variation in the promoter of *qRBG1/OsBZR5* enhanced rice yield by *OsGSK2*–*qRBG1*–*OsBZR1*–*D2*–*OFP1* in the Brassinolide signaling pathway (Zhang et al., 2024b). Through cloning and further study of related genes by an increasing number of researchers, our understanding of the molecular mechanism underlying rice yield formation has gradually established and deepened. It has also laid a good foundation for researching and developing molecular breeding for rice yield and quality. In addition, there is a growing awareness of the complexity and multifaceted nature of various genes and metabolic pathways in controlling the traits of rice (Lei et al., 2024). At present, although some QTLs for yield-related traits in rice have been identified, their number remains insufficient to fulfill the requirement (Wang et al., 2021). Especially because some of these genes are identified from mutants, which often pose serious challenges such as reduced plant height and reduced seed setting ratio, it is more difficult to be directly applied as breeding materials in breeding practice. However, using CSSLs, especially single-segment substitution lines (SSSLs), it is possible to find alleles of these cloned genes in them and identify new favorable genes for yield and quality traits. Moreover, CSSLs are excellent breeding materials for gene identification and for the pyramid of favorable genes (Balakrishnan et al., 2018).

In this study, we aimed to construct an *indica* SSSL library covering the entire rice genome and to identify QTLs for yield-related traits in rice. We used the rice CSSL-Z691 containing four substitution segments from the *indica* rice restoration line Jinhui35 in the genetic background of Xihui18 as experimental materials. We used the secondary F_2_ population constructed from the cross between Xihui18 and Z691 for the QTL mapping of important agronomic traits in rice. We further built three SSSLs and three dual-segment substitution lines (DSSLs) by marker-assisted selection (MAS). Then, we used the SSSLs and DSSLs for further QTL identification and the analysis of the epistatic effect between different QTLs. In addition, we constructed the fine-mapping population and the overlapping substitution segment mapping population of *qGL4* and *qGW6* and accordingly completed the fine-mapping of *qGL4* and *qGW6*. The results of this study provide a foundation for map-based cloning of QTLs and for “breeding by design” in rice.

## 2 Materials and methods

### 2.1 Experimental materials

#### 2.1.1 Development of the short-width rice CSSL-Z691

Z691 is a four-segment CSSL selected by MAS from the progeny (F_2:4_) of a cross between Xihui18 and Z403. Xihui18 is an *indica* rice restorer line bred by Southwest University, China. Z403 is a CSSL containing 12 segments from the *indica* rice restorer line Jinhui35 on the genetic background of Xihui18. The flowchart of Z691 development is shown in **Supplementary Figure S1**. The specific development method of Z403 was described by Xu et al. (2023). The substitution lengths of Z691 were estimated by the method of Paterson et al. (1991), and chromosome substitution segments were mapped using the MapChart 2.32 software (https://www.wur.nl/en/show/Mapchart.htm).

#### 2.1.2 Materials for QTL mapping

For the QTL mapping, a secondary F_2_ segregating population of 150 individuals raised from the cross between Xihui18 and Z691 was used.

#### 2.1.3 Materials for the development of SSSLs and DSSLs

Six F_2_ individuals with the target substitution marker and the other 0–1 heterozygous marker were screened according to the result of QTL mapping to develop SSSLs and DSSLs.

#### 2.1.4 Materials for fine-mapping of *qGL4* and *qGW6*

The fine-mapping population of *qGL4* was an F_3_ population with 631 individuals. These individuals were developed from a recombinant plant of *qGL4* in the F_2_ population. Additionally, four individuals with different genotypes were screened from the F_3_ population to establish an overlapping substitution mapping population of *qGL4*.

The fine-mapping population of *qGW6* was an F_3_ population with 100 individuals. These individuals were developed from a recombinant plant of *qGW6* in the F_2_ population. Two individuals with different genotypes were screened from the F_3_ population to develop an overlapping substitution mapping population of *qGW6*.

### 2.2 Experimental methods

#### 2.2.1 Field planting

In July 2020, Xihui18 was crossed with Z691 to obtain hybrid seeds at the Chongqing experimental station of Southwest University of China (29.76°N, 106.38°E). In September of the same year, the hybrid seeds were planted to obtain F_1_ seeds at the Lingshui experimental base in Hainan province, China (18.42°N, 109.8°E). In March 2021, Xihui18, Z691, and the F_2_ population were planted at the experimental station of the Southwest University of Chongqing, China. In April, 30 plants of Xihui18 and Z691 and 150 individuals of the F_2_ population were transplanted in the same field. The space between plants was 16.80 cm, and that between rows was 26.50 cm. In March 2022, Xihui18, Z691, and six F_2_ individuals for developing SSSLs and DSSLs were transplanted, with 30 plants for each material, on April 15, 2022, together with 631 individuals for *qGL4* fine-mapping and 100 individuals for *qGW6* fine-mapping. In March 2023, Xihui18, Z691, three SSSLs (S1–S3), and three DSSLs (D1–D3) were planted in the same experimental field, and 30 plants were transplanted for each material in April of the same year. In addition, six secondary heterozygous F_3_ individuals for developing SSSLs were planted for overlapping substitution mapping of *qGL4* and *qGW6*, each line with 100 individuals. Conventional field management practices were applied (according to the local rice management methods of the two experimental bases; the experimental fields used are representative of the local areas).

#### 2.2.2 Measurement of yield-related traits

Ten plants each from Xihui18, Z691, SSSLs, and DSSLs plots, together with 150 F_2_ plants, were harvested at the maturity stage. Twelve yield-related traits were measured, including plant height, spikelet number per panicle, panicle length, number of primary panicles, number of secondary panicles, grain number per panicle, seed setting ratio, seed-set density, length of 10 grains, width of 10 grains, and 1000-grain weight. The measurement methods were the same as those of Ma et al. (2020). Panicle length, spikelet number per panicle, grain number per panicle, and seed setting ratio were measured using all the effective panicles of the plant. In this case, the seed setting ratio was expressed as the percentage of grain number per panicle to the spikelet number per panicle. The seed-set density was expressed as the mean of the total number of grains per 10 cm panicle length. The length and width of 10 grains for each plant were measured using a vernier caliper, and then the mean values were calculated. According to the mean value of the length and width of 10 grains, the rate of grain length to width was obtained for each plant. The 1000-grain weight of Xihui18 and Z691 were measured using an electronic balance, with three repetitions. The 1000-grain weight of each F_2_ plant was determined as the weight of 200 grains multiplied by 5, with three repetitions. Yield per plant was weighed using an electronic balance. Finally, various parameters such as minimum, maximum, mean, and standard deviation of the above agronomic trait measurements of 10 plants of Xihui18, Z691, and 150 F_2_ populations were counted and underwent a t-test using Microsoft Excel 2010.

#### 2.2.3 QTL mapping

A secondary F_2_ population with 150 individuals was used for QTL mapping. They were derived from a cross between Xihui18 and Z691. The total genomic DNA of Xihui18, Jinhui35, Z691, and the 150 individuals from the F_2_ population was extracted using the cetyltrimethylammonium bromide method (McCouch et al., 1988). PCR amplification, non-denaturing polyacrylamide gel electrophoresis, and rapid silver staining were conducted using the methods described by Zhao et al. (2016). Bands identical to Xihui18 received a score of “−1”, bands identical to Z691 received a score of “1”, heterozygous bands received a score of “0”, and the lack of marker bands received a score of “.”. For QTL mapping, the phenotypic values of each member of the F_2_ population and the marker assignments were utilized. QTL mapping was performed using the restricted maximum likelihood method by mixed linear models (REML) implemented in the HPMIXED procedure of SAS 9.3 (SAS Institute Inc., Cary, NC, USA), with significance determined at α = 0.05 (Zhao et al., 2016; Sun et al., 2022).

#### 2.2.4 Development of SSSLs and DSSLs, and verification and pyramid analysis of QTLs

Combining the results of phenotypic analysis and preliminary QTL localization, six individual plants containing the target QTLs and 0–1 heterozygous markers were selected from the F_2_ population and planted as lines in the same field in 2022. Ten individual plants were taken from each line and further characterized using MAS to select single-segment substitution lines and dual-segment substitution lines containing the target QTLs.

In the fall of 2023, 10 plants of Xihui18 and SSSLs (S1–S3) were harvested to measure the above yield-related traits. As there was only one chromosome segment difference between each SSSL and Xihui18 under the same environmental conditions, it can be assumed that all the genetic traits exhibited by SSSLs that were different from Xihui18 were associated with this differential chromosome segment. Therefore, the genetic model of the recipient parent Xihui18 was *P*_0_ = *μ*_0_ + *ϵ*, and the genetic model of SSSLs carrying specific QTLs was *P_i_* = *μ*_0_ + *a_i_* + *ϵ*, where *μ*_0_ is the average of the Xihui18 population phenotypes, *a_i_* is the additive effect of the QTLs; *p_i_* and *p*_0_ represent the phenotypic values of the SSSLs and Xihui18, respectively; and *ϵ* is the random error. Each SSSL_i_ (S1, S2, and S3) was hypersized (H0) to have no QTLs in the substituted segments of SSSL_i_. One-way ANOVA and Duncan’s multiple comparisons in IBM SPSS Statistics 23.0 were used to statistically analyze each trait for each SSSL_i_ with 10 plants of SSSL_i_ and Xihui18, and QTL was considered present in SSSL_i_ when the *p*-value was <0.05. Therefore, the additive effect of the QTL can be calculated as *a_i_* = (*p_i_* − *p*_0_)/2 (one-half of the phenotypic difference was considered to be caused by genetics) (Li et al., 2022a).

Similarly, 10 plants of Xihui18 and DSSL (D1–D3) were harvested to measure the above yield-related traits in the fall of 2023. In the same environment, the genetic model of DSSL was *P_ij_* = *μ*_0_ + *a_i_* + *a_j_* + *I_ij_* + *ϵ*, where *P_ij_* represents the phenotypic value of DSSL_ij_, *a_i_* and *a_j_* represent the additive effect of QTL in substitution segment *i* and *j*, respectively, and *I_ij_* represents the *a_i_a_j_* epistasis effect between QTLs in substitution segment *i* and *j*. With this, two-way ANOVA and Duncan’s multiple comparisons in IBM SPSS Statistics 23.0 were utilized to analyze the significance between Xihui18, DSSL_ij_, SSSL_i_, and SSSL_j_ for each trait, where Xihui18, SSSL_i_, SSSL_j_, and DSSL_ij_ represent the phenotypic values of the corresponding phenotypic traits of Xihui18, SSSL, and DSSL, respectively. When the *p*-value was >0.05, it was considered that two QTLs were inherited independently. When the probability value was less than 0.05, it was considered that there was an epistatic effect among QTLs. Therefore, the epistatic effect between non-equivalent QTLs was estimated as [(Xihui18 + DSSL_ij_) − (SSSL_i_ + SSSL_j_)]/2 (one-half of the phenotypic difference was considered to be caused by genetics) (Li et al., 2022a; Liang et al., 2021).

#### 2.2.5 Fine-mapping and overlapping substitution mapping of the major QTLs for grain length—*qGL4*

Based on the *qGL4* primary mapping, an F_3_ population of 631 plants constructed by one recombinant line of qGL4 self-fertilization was used for genetic analysis and fine-mapping of *qGL4* in 2022. Among them, 151 short-grain recessive individual plants, three newly synthesized polymorphic seed setting ratio (SSR) markers, and one polymorphic SSR marker in the initial localization of *qGL4* were used for linkage analysis. In addition, four single plants with different genotypes were selected to breed secondary SSSLs of *qGL4* in the F_3_ population in 2023. All selected SSSLs and Xihui18 were then measured for grain length and used for overlapping substitution mapping of *qGL4*. When the grain lengths of secondary SSSLs and Xihui18 were significantly different, it indicated that a QTL for grain length was detected in the substitution segments of SSSLs. The QTL was localized in the overlapping region when multiple substitution segments with the target trait overlapped (Yang et al., 2021). Then, within the interval of *qGL4*, the candidate genes were predicted according to the possible gene family reported by Li et al. (2018) by the Gramene (https://www.gramene.org/), the Rice Annotation Project (https://rapdb.dna.affrc.go.jp/), and China Rice Data Center (http://www.ricedata.cn/gene/index.htm) databases; the entire DNA of these genes between S1 and Xihui18 was sequenced.

#### 2.2.6 Overlapping substitution mapping of the major QTLs for grain width—*qGW6*

Based on the initial mapping of *qGW6*, in 2022, an F_3_ population consisting of 100 individuals was constructed from the self-fertilization of a recombinant plant of *qGW6*. Subsequently, polymorphic markers were synthesized within this interval, and the existing substitution markers were used to conduct MAS on the 100 individual plants. This process aimed to build secondary SSSLs with different genotypes at this locus (some markers were not homozygous). In 2023, 100 plants of each of the selected non-homozygous SSSLs were planted to continue the construction of homozygous SSSLs. Subsequently, all the bred SSSLs with the same genotype and Xihui18 were measured for grain width to be used for the overlapping substitution mapping of *qGW6*. Then, within the interval of *qGW6*, the candidate genes were predicted by the related websites involved above, and the entire DNA of these genes between S2 and Xihui18 was sequenced.

## 3 Results

### 3.1 Identification of substitution segments of Z691

**Figure 1:**
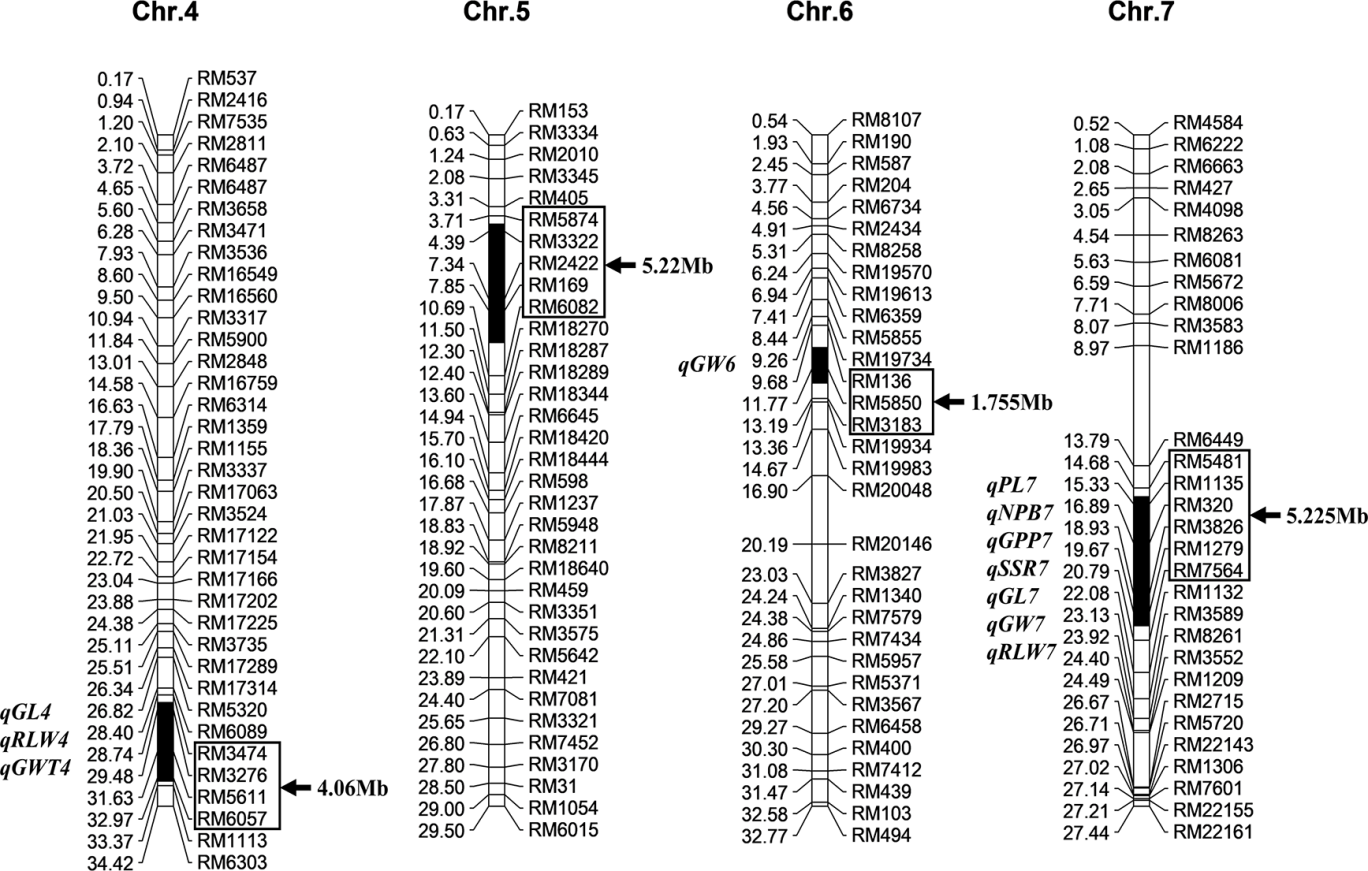
Substitution segments and detected quantitative trait loci (QTLs) of Z691. The left side of each chromosome is the physical distance (Mb) and the QTL of positioning; the right side is the marker name and the length of the chromosome replacement fragment. Substitution intervals (in-box markers). GL is the grain length, RLW is the rate of length to width, GWT is 1000-grain weight, GW is the grain width, PL is panicle length, NPB is number of primary branches, GPP is grain number per panicle, and SSR is seed setting ratio.

According to the previous identification of Z691 substitution segments with 236 polymorphic SSR markers between two parents screened from 429 markers covering the whole rice genome, 10 Z691 plants were further identified with 10 SSR markers on the Z691 substitution segment and more than 90 SSR markers outside the substitution segment for the further identification of substitution segments and genetic background purity testing. The results showed that the substitution segments of 10 plants of Z691 were identical, and no other residual segments from Jinhui35 were detected. This indicates that the genotype of Z691 has become homozygous. Z691 contained four substitution segments from Jinhui35, distributed on chromosomes 4, 5, 6, and 7. The total length of these substitution segments in Z691 was estimated as 16.26 Mb. Among them, the longest substitution segment on Z691 was estimated as 5.225 Mb, the shortest one was 1.755 Mb, and the average length of these substitution segments was 4.065 Mb (**Figure 1**).

### 3.2 Analysis of important agronomic traits of Z691

**Figure 2:**
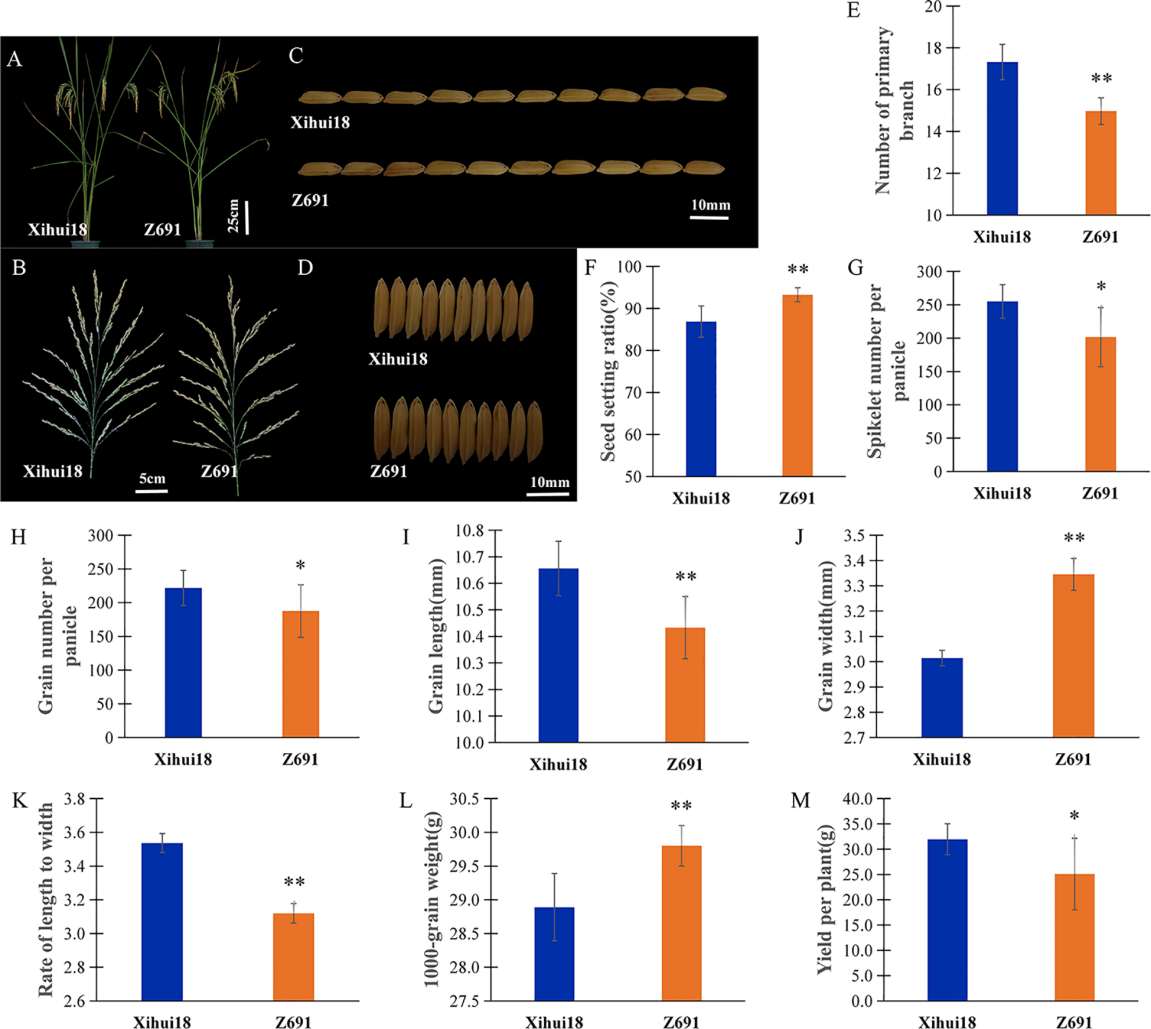
Phenotypic and differential traits of CSSL-Z691 and its recipient Xihui18. **(A)** Plant type of Xihui18 (left) and Z691 (right). **(B)** Main panicle of Xihui18 (left) and Z691 (right). **(C)** Grain length of Xihui18 (upper)and Z691 (lower). **(D)** Grain width of Xihui18 (upper) and Z691 (lower). **(E)** Number of primary branches per plant. **(F)** Seed setting ratio. **(G)** Spikelet number per panicle. **(H)** Grain number per panicle. **(I)** Grain length. **(J)** Grain width. **(K)** Rate of length to width. **(L)** 1000-grain weight. **(M)** Yield per plant of Xihui18 and Z691. Data are given as the mean and SE (n = 10). * and ** indicate significant differences of traits between Xihui18 and Z691 at *p* < 0.05 and *p* < 0.01, respectively. Bars in **(A)**, 25 cm; **(B)**, 5 cm; **(C, D)**, 10 mm.

Z691 had a plant type similar to the recipient parent, Xihui18. However, there were still many traits that were different; the heading date of Z691 displayed earlier 5 d than that (115 d) of Xihui18 in Chongqing, China (**Figure 2A**). Compared to Xihui18, Z691 showed a significant increase in seed setting ratio (93.26%) (**Figures 2B, F**), grain width (3.35 mm) (**Figures 2D, J**), and 1000-grain weight (29.80 g) (**Figure 2L**), with increases of 7.35%, 11.30%, and 3.15%, respectively. However, Z691 exhibited a significant decrease in the number of primary branches (14.98) (**Figure 2E**), spikelet number per panicle (201.57 grains) (**Figure 2G**), grain number per panicle (187.48 grains) (**Figure 2H**), grain length (10.43 mm) (**Figures 2C, I**), the rate of length to width (3.12) (**Figure 2K**), and yield per plant (25.09 g) (**Figure 2M**), with reductions of 13.51%, 21.18%, 18.29%, 2.16%, 11.86%, and 21.52%, respectively.

### 3.3 QTL mapping for important agronomic traits in the secondary F_2_ population of Xihui18/Z691

Eleven QTLs from Jinhui35 were detected in the secondary F_2_ population from a cross between Xihui18 and Z691. These QTLs were distributed on three substitution segments of Z691 and explained the phenotypic variation from 3.72% to 46.43% (**Table 1**). Among them was one QTL for panicle length, number of primary branches, grain number per panicle, grain weight, and seed setting ratio. Moreover, there were two QTLs for grain length, grain width, and the rate of length to width. Except for *qGW7*, the other were all major QTLs, explaining more than 10% of the variation. The additive effect of *qPL7* reduced the panicle length of Z691 by 0.65 cm, explaining 11.66% of the variation in the panicle length. The additive effect of *qNPB7* decreased the number of primary branches of Z691 by 0.70, explaining 35.29% of the variation in the number of primary branches. The additive effect of the *qGPP7* reduced the grain number per panicle of Z691 by 14.23 grains, explaining 11.22% of the variation in grain number per panicle. The additive effect of the *qSSR7* decreased the seed setting ratio of Z691 by 3.85%, explaining 19.27% of the variation in the seed setting ratio. *qGL4* and *qGL7-1* were major QTLs, controlling the grain length of Z691, and their additive effects reduced the grain length of Z691 by 0.16 and 0.20 mm, respectively. *qGL4* and *qGL7-1* explained 22.14% and 34.39% of the variation in grain length, respectively. The grain width of Z691 was mainly controlled by *qGW6* and *qGW7*, whose additive effects increased the grain width of Z691 by 0.03 mm and decreased it by 0.02 mm, respectively. *qGW6* and *qGW7* explained 10.61% and 3.72% of the variation of grain length, respectively. The additive effects of *qRLW4* and *qRLW7* reduced the rate of length to width of Z691 by 0.05 and 0.08, respectively. *qRLW4* and *qRLW7* explained 18.93% and 46.43% of the variation in the rate of length to width, respectively. The additive effect of *qGWT4* on the 1000-grain weight of Z691 could reduce the 1000-grain weight of Z691 by 0.75 g, explaining 16.72% of the variation of 1000-grain weight (**Table 1**).

**Table 1:** QTL for rice important traits carried by substitution segment on Z691.

Trait	QTL	Chr.	Linked marker	Additive effect	Var. (%)	*p*-Value	Possible candidate genes
Panicle length (cm)	*qPL7*	7	RM1135	−0.65	11.66	0.0347	
Number of primary branches	*qNPB7*	7	RM1135	−0.70	35.29	0.0018	
Grain number per panicle	*qGPP7*	7	RM1135	−14.23	11.22	0.0372	*OsHXK1*
Seed setting ratio (%)	*qSSR7*	7	RM1135	−3.85	19.27	0.0131	*OsHXK1*
Grain length (mm)	*qGL4*	4	RM3276	−0.16	22.14	0.0001	*FLO2*; *DHT1*
	*qGL7-1*	7	RM1135	−0.20	34.39	0.0029	
Grain width (mm)	*qGW6*	6	RM5850	0.03	10.61	0.0276	*OsGSR1*
	*qGW7*	7	RM1279	−0.02	3.72	0.0355	*OsBZR1*
Rate of length to width	*qRLW4*	4	RM3276	−0.05	18.93	0.0003	*FLO2*; *DHT1*
	*qRLW7*	7	RM1135	−0.08	46.43	0.0006	
1000-grain weight (g)	*qGWT4*	4	RM3276	−0.75	16.72	0.0007	*FLO2*; *DHT1*

QTLs, quantitative trait loci.

### 3.4 Development of SSSLs and DSSLs, as well as validation and interaction analysis of QTLs using the SSSLs and DSSLs

**Figure 3:**
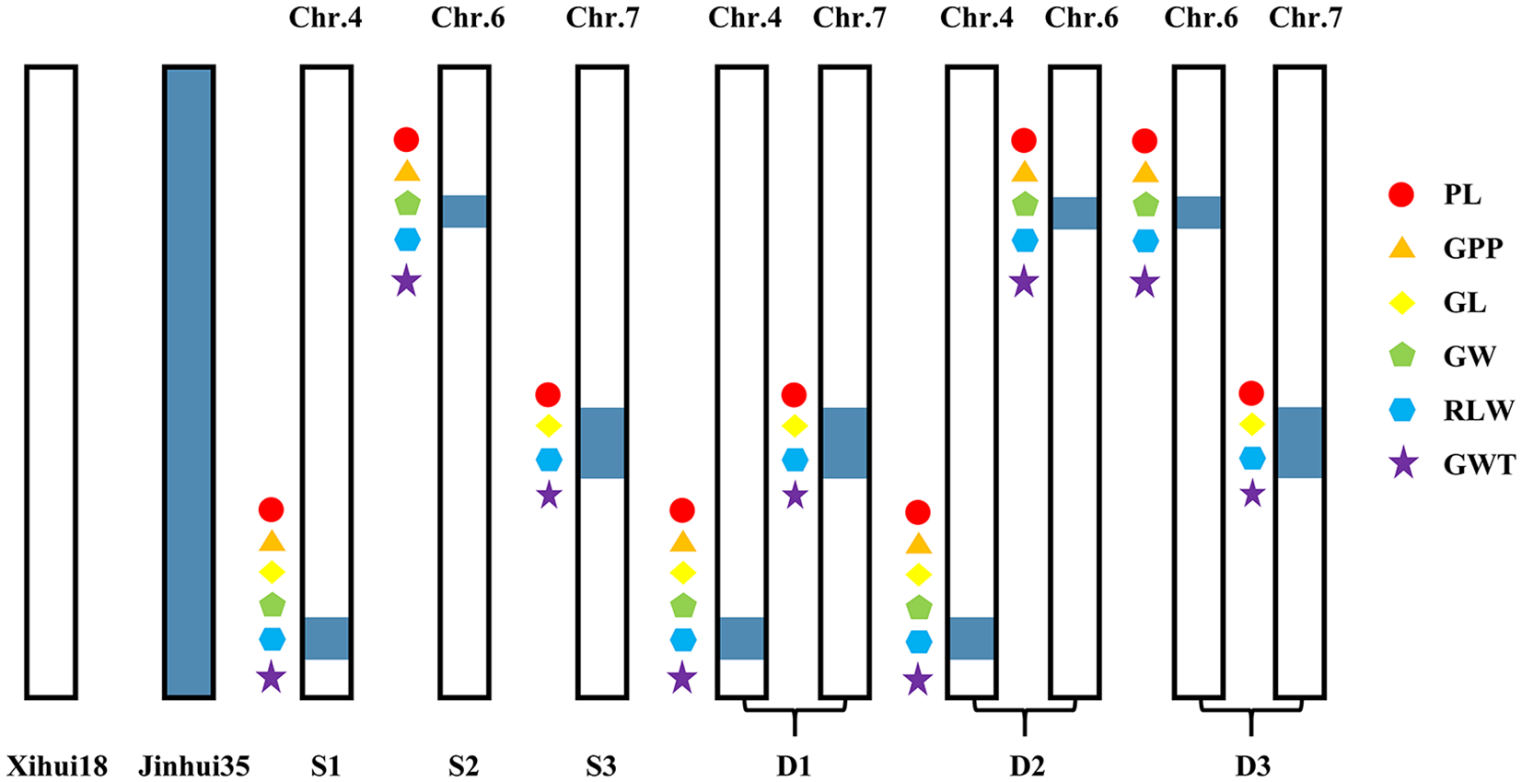
Map of substitution segment on secondary substitution lines. Chr, chromosome; S, single-segment substitution line; D, dual-segment substitution line. PL is panicle length, GPP is grain number per panicle, GL is the grain length, GW is the grain width, RLW is the rate of length to width, and GWT is the 1000-grain weight. S1: [Chr.4:RM3474 (28.74 Mb)-RM3276 (29.48 Mb)-RM5611 (31.63 Mb)–RM6057 (32.97 Mb)]. S2: [Chr.6:RM136 (9.68 Mb)–RM5850 (11.77 Mb)–RM3183 (13.19 Mb)]. S3: [Chr.7:RM3826 (18.93 Mb)–RM1279 (19.67 Mb)–RM7564 (20.79 Mb)]. D1: [Chr.4:RM3474 (28.74 Mb)-RM3276 (29.48 Mb)-RM5611 (31.63 Mb)–RM6057 (32.97 Mb), Chr.7:RM3826 (18.93 Mb)–RM1279 (19.67 Mb)–RM7564 (20.79 Mb)]. D2: [Chr.4:RM3474 (28.74 Mb)-RM3276 (29.48 Mb)-RM5611 (31.63 Mb)–RM6057 (32.97 Mb), Chr.6:RM136 (9.68 Mb)–RM5850 (11.77 Mb)–RM3183 (13.19 Mb)]. D3: [Chr.6:RM136 (9.68 Mb)–RM5850 (11.77 Mb)–RM3183 (13.19 Mb), Chr.7:RM3826 (18.93 Mb)–RM1279 (19.67 Mb)–RM7564 (20.79 Mb)]. Internal tags connected with “-” indicate substitution fragments from donors, while tags at both ends of substitution fragments connected with “–” indicate that fragment reorganization may occur.

Based on the QTL mapping results, the MAS was utilized in the F_3:4_ generation to dissect further three SSSLs (S1–S3) and three DSSLs (D1–D3). S1 harbored the substitution segment RM3474–RM3276-RM5611–RM6057 of chromosome 4. S2 harbored the substitution segment RM136–RM5850–RM3183 of chromosome 6. S3 harbored the substitution segment RM3826–RM1279–RM7564 of chromosome 7. D1 harbored one substitution segment on chromosome 4 (RM3474–RM3276-RM5611–RM6057) and one substitution segment on chromosome 7 (RM3826–RM1279–RM7564). D2 harbored one substitution segment on chromosome 4 (RM3474–RM3276-RM5611–RM6057) and one substitution segment on chromosome 6 (RM136–RM5850–RM3183). D3 harbored one substitution segment on chromosome 6 (RM136–RM5850–RM3183) and one substitution segment on chromosome 7 (RM3826–RM1279–RM7564). All these substitution segments above were from Jinhui35 (**Figure 3**).

**Figure 4:**
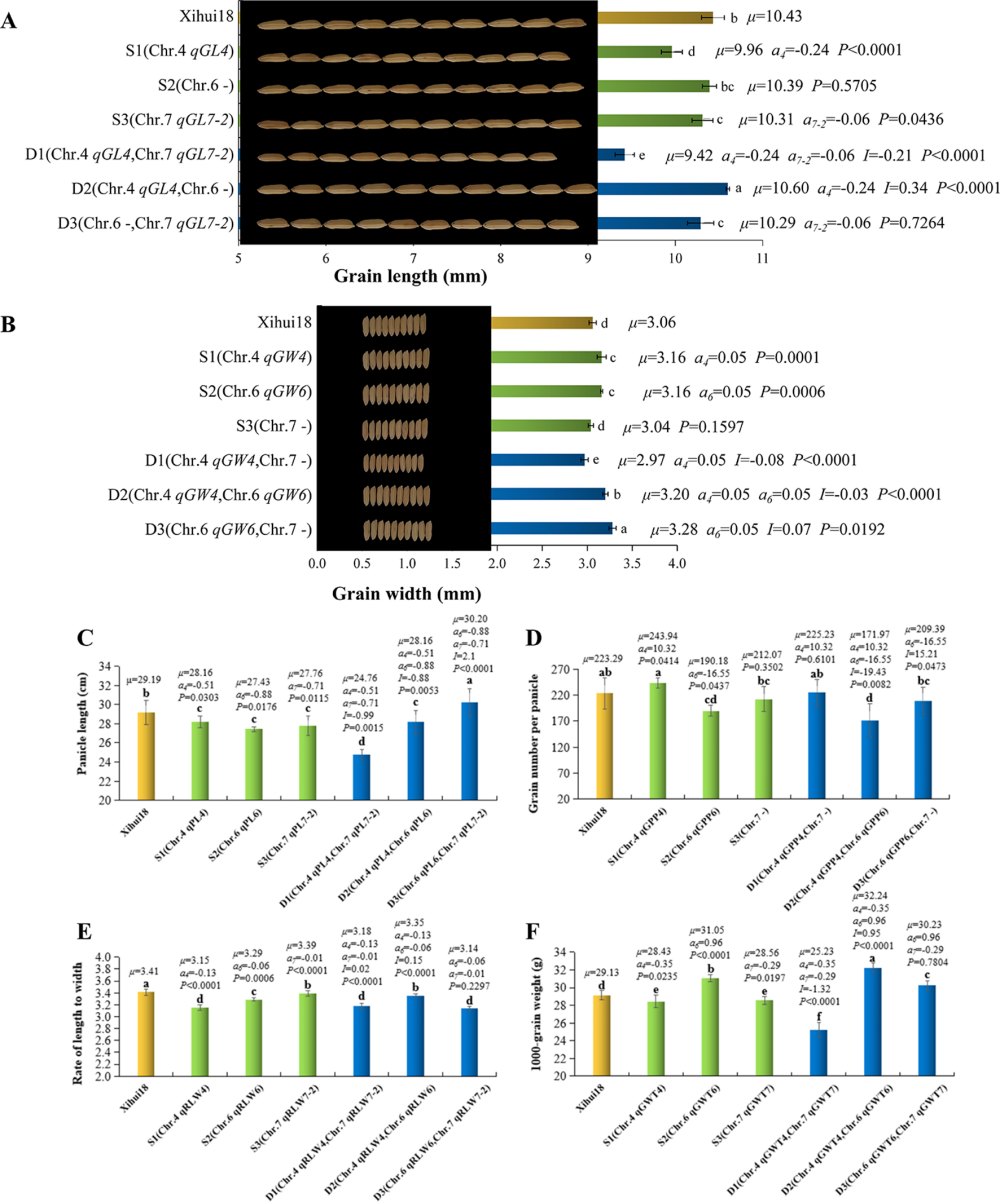
Analysis of additive and epistatic effects of quantitative trait loci (QTLs) for related traits of single-segment substitution lines (SSSLs) and dual-segment substitution lines (DSSLs). **(A)** Grain length (GL). **(B)** Grain width (GW). **(C)** Panicle length (PL). **(D)** Grain number per panicle (GPP). **(E)** Rate of length to width (RLW). **(F)** 1000-grain weight (GWT). The *p*-value of SSSL indicates the probability of a significant difference between SSSL and Xihui18 by one-way ANOVA, and *p* < 0.05 denotes carrying QTL in the substitution segment. The *p*-value of DSSL represents the probability of QTL_1_ × QTL_2_ of the DSSL, and *p* < 0.05 denotes existing epistatic effects between QTL_1_ and QTL_2_ of DSSL. S1: [Chr.4:RM3474 (28.74 Mb)-RM3276 (29.48 Mb)-RM5611 (31.63 Mb)–RM6057 (32.97 Mb)]. S2: [Chr.6:RM136 (9.68 Mb)–RM5850 (11.77 Mb)–RM3183 (13.19 Mb)]. S3: [Chr.7:RM3826 (18.93 Mb)–RM1279 (19.67 Mb)–RM7564 (20.79 Mb)]. D1: [Chr.4:RM3474 (28.74 Mb)-RM3276 (29.48 Mb)-RM5611 (31.63 Mb)–RM6057 (32.97 Mb), Chr.7:RM3826 (18.93 Mb)–RM1279 (19.67 Mb)–RM7564 (20.79 Mb)]. D2: [Chr.4:RM3474 (28.74 Mb)-RM3276 (29.48 Mb)-RM5611 (31.63 Mb)–RM6057 (32.97 Mb), Chr.6:RM136 (9.68 Mb)–RM5850 (11.77 Mb)–RM3183 (13.19 Mb)]. D3: [Chr.6:RM136 (9.68 Mb)–RM5850 (11.77 Mb)–RM3183 (13.19 Mb), Chr.7:RM3826 (18.93 Mb)–RM1279 (19.67 Mb)–RM7564 (20.79 Mb)). Internal tags connected with “-” indicate substitution fragments from donors, while tags at both ends of substitution fragments connected with “–” indicate that fragment reorganization may occur. Different small letters indicate a significant difference (P <0.05) as determined by Duncan’s multiple comparisons.

**Figure 5:**
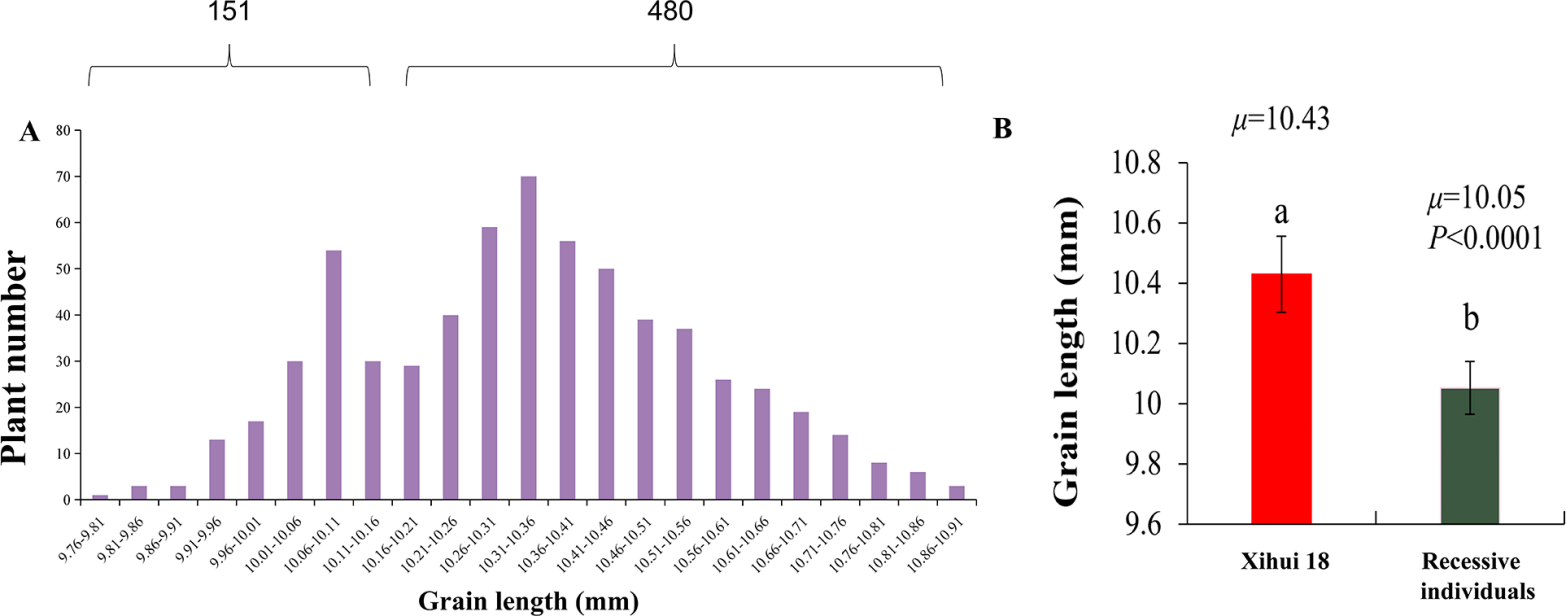
Genetic analysis of grain length of *qGL4*. **(A)** Distribution of grain length in F_3_ population. **(B)** Comparison of grain length between Xihui18 and recessive individuals. Different small letters indicated the sigmificant difference between Xihui18 and the recessive individuals.

S1 contained the substitution segment on chromosome 4. A total of six QTLs (*qGL4*, *qRLW4*, *qGWT4*, *qPL4*, *qGPP4*, and *qGW4*) were detected in S1 (**Figure 3**). Among them, *qGL4*, *qRLW4*, and *qGWT4* can also be detected in the F_2_ population, with their additive effects being −0.24 mm (**Figure 4A**), −0.13 (**Figure 5E**), and −0.35 g (**Figure 4F**), respectively. Additionally, three new QTLs, *qPL4*, *qGPP4*, and *qGW4*, were detected in S3, with their additive effects being −0.51 cm (**Figure 4C**), 10.32 grains (**Figure 4D**), and 0.05 mm (**Figure 4B**), respectively. However, these QTLs were not detected in the F_2_ population.

S2 contained the substitution segment on chromosome 6. A total of five QTLs (*qGW6*, *qPL6*, *qGPP6*, *qRLW6*, and *qGWT6*) were detected in S2 (**Figure 3**). Among these, *qGW6* can also be detected in the F_2_ population, with an additive effect of 0.05 mm (**Figure 4E**). In addition, four new QTLs, *qPL6*, *qGPP6*, *qRLW6*, and *qGWT6*, were detected in S4, with additive effects being −0.88 cm (**Figure 4C**), 16.55 grains (**Figure 4D**), −0.06 (**Figure 4E**), and 0.96 g (**Figure 4F**), respectively.

S3 contained the substitution segment on chromosome 7. A total of four QTLs (*qGW7*, *qPL7-2*, *qGL7-2*, *qRLW7-2*, and *qGWT7*) were detected in S3 (**Figure 3**). Among them, *qGW7*, which was detected in the F_2_ population, was not confirmed by S3. However, four new QTLs, *qPL7-2*, *qGL7-2*, *qRLW7-2*, and *qGWT7*, were detected in S5, with additive effects being −0.71 cm (**Figure 4C**), −0.06 mm (**Figure 4A**), −0.01 (**Figure 4E**), and −0.29 g (**Figure 4F**), respectively.

In conclusion, among the 11 QTLs (*qPL7*, *qNPB7*, *qGPP7*, *qSSR7*, *qGL4*, *qGL7*, *qGW6*, *qGW7*, *qRLW4*, *qRLW7*, and *qGWT4*) detected in the secondary F_2_ population of Xihui18/Jinhui35, four QTLs (*qGL4*, *qRLW4*, *qGWT4*, and *qGW6*) were validated by their corresponding SSSLs, with only the minor effect QTL, *qGW7*, not confirmed by its corresponding SSSL. The other six QTLs, *qPL7*, *qNPB7*, *qGPP7*, *qSSR7*, *qGL7*, and *qRLW7*, could not be validated due to the absence of corresponding SSSLs. Therefore, these six QTLs require further validation in the following years. In addition, within these three SSSLs (S1–S3), 11 new QTLs (*qPL4*, *qGPP4*, *qGW4*, *qPL6*, *qGPP6*, *qRLW6*, *qGWT6*, *qPL7-2*, *qGL7-2*, *qRLW7-2*, and *qGWT7*) were detected. These QTLs were not identified in the F_2_ population but in the corresponding SSSLs, suggesting that SSSLs offer a higher efficiency in QTL detection due to only one segment on Jinhui35 introgressed in each SSSL based on the Xihui18 background, eliminating the influence of other identical trait QTLs (Deng et al., 2023).

The interaction of *qGL4* (*a* = −0.24 mm) and *qGL7-2* (*a* = −0.06 mm) produced an epistatic effect of −0.21, which decreased grain length genetically by 0.51 mm in D1. Owing to −0.51 < −0.24 < −0.06, the grain length (9.42 mm) of D1 was significantly shorter than that (9.96, 10.31, and 10.43 mm) of S1 (*qGL4*), S3 (*qGL7-2*), and Xihui18. The interaction of *qGL4* (*a* = −0.24 mm) and a substitution locus without GL on chromosome 6 in D2 produced an epistatic effect of 0.34, which increased grain length genetically by 0.10 mm. Thus, the grain length (10.60 mm) of D2 was significantly longer than that (9.96, 10.39, and 10.43 mm) of S1 (*qGL4*), S2 (without GL QTL), and Xihui18. This indicates that the substituted segment on chromosome 6, which does not contain grain length QTLs, also significantly affects the expression of *qGL4*, resulting in a significant increase in grain length in D2. However, *qGL7-2* (*a* = −0.06 mm) and a substitution locus without GL on chromosome 6 belonged to independent inheritance in D3, indicating that the genetic effect of D3 is equal to the additive effect (−0.06 mm) of *qGL7-2*. Accordingly, the grain length (10.29 mm) of D3 displayed no significant difference compared to that (10.31 mm) of S3 containing *qGL7-2* while significantly shorter than that (10.39 and 10.43 mm) of S2 and Xihui18 (**Figure 4A**).

The interaction of *qGW4* (*a* = 0.05 mm) and a substitution locus without grain width (GW) on chromosome 7 in D1 produced an epistatic effect of −0.08, resulting in −0.03 mm of the genetic effect of D1 for grain width. Thus, the grain width (2.97 mm) of D1 was significantly narrower than that (3.16, 3.04, and 3.06 mm) of S1 (*qGW4*), S3 (without GW QTL), and Xihui18. This indicates that the substitution segment on chromosome 7, no grain width QTL, also significantly affects the expression of *qGW4*, resulting in a significant decrease in grain length in D1. The interaction of *qGW4* (*a* = 0.05 mm) and *qGW6* (*a* = 0.05 mm) produced an epistatic effect of −0.03, causing the genetic effect of 0.07 mm in D2, larger than any of the additive effects of the single QTL. Accordingly, the grain width (3.20 mm) of D2 was significantly wider than that (3.16, 3.16, and 3.06 mm) of S1 (*qGW4*), S2 (*qGW6*), and Xihui18. This indicates that the interaction of *qGW4* and *qGW6* creates a wider grain. The interaction of *qGW6* (*a* = 0.05 mm) and a substitution locus without GW on chromosome 7 produced an epistatic effect of 0.07, which increased GW genetically by 0.12 mm in D3, larger than the additive effect of *qGW6*. Thus, the grain width (3.28 mm) of D3 was significantly broader than that (3.16, 3.04, and 3.06 mm) of S2 (*qGW6*), S3 (without GW QTL), and Xihui18. This indicates that the substituted segment on chromosome 7, no GW QTL, also significantly affects the expression of *qGW6*, resulting in a significant increase in grain width in D3 (**Figure 4B**).

The interaction of *qPL4* (*a* = −0.51 cm) and *qPL7-2* (*a* = −0.71 cm) produced an epistatic effect of −0.99 cm, resulting in −2.21 cm of the genetic effect of D1 for panicle length, less than any of the additive effect of each single QTL. Thus, the panicle length (24.76 cm) of D1 was significantly shorter than that (28.16, 27.76, and 29.19 cm) of S1 (*qPL4*), S3 (*qPL7-2*), and Xihui18. This indicates that the interaction of *qPL4* and *qPL7-2* resulted in a shorter panicle length. The interaction of *qPL4* (*a* = −0.51 cm) and *qPL6* (*a* = −0.88 cm) produced an epistatic effect of 0.88 cm, resulting in −0.51 cm of genetic effect for panicle length in D2, equal to the additive effects of *qPL4* and *qPL6*. Thus, the panicle length (28.16 mm) of D2 displayed no significant difference compared to that (28.16 and 27.43 cm) of S1 (*qPL4*) and S2 (*qPL6*) while significantly shorter than that (3.06 mm) of Xihui18. The interaction of *qPL6* (*a* = −0.88 cm) and *qPL7-2* (*a* = −0.71 cm) produced an epistatic effect of 2.10 cm, causing 0.51 cm of the genetic effect for panicle length in D3, larger than all the additive effects (−0.88 and −0.71 cm) of each single QTL. Accordingly, the panicle length (30.20 cm) of D3 was significantly longer than that (27.43, 27.76, and 29.19 cm) of S2 (*qPL6*), S3 (*qPL7-2*), and Xihui18 (**Figure 4C**). This indicates that the pyramiding of *qPL6* and *qPL7-2* can produce a longer panicle length.

*qGPP4* (*a* = 10.32 grains) and a substitution locus without grain number per panicle (GPP) on chromosome 7 in D1 belonged to independent inheritance. Thus, the genetic effect for grain number per panicle of D1 equals the additive effect of *qGPP4*. Accordingly, D1 displayed no significant difference in grain number per panicle (225.23 grains) from S1 (243.94 grains) containing *qGPP4*. The interaction of *qGPP4* (*a* = 10.32 grains) and *qGPP6* (*a* = −16.55 grains) produced an epistatic effect of −19.43 grains, resulting in −25.66 grains of genetic effect in D2, parallel to the additive effect of *qGPP6*. Thus, the grain number per panicle (171.97 grains) of D2 exhibited no significant difference from that (190.18 grains) of S2 carrying *qGPP6* while significantly less than that (243.94 and 223.29 grains) of S1 (*qGPP4*) and Xihui18. This indicates that *qGPP6* is epistatic to *qGPP4*. The interaction of *qGPP6* (*a* = −16.55 grains) and a substitution locus without GPP on chromosome 7 produced an epistatic effect of 15.21 grains, resulting in −1.34 grains of the genetic effect of D3, parallel to the additive effect of *qGPP6*. Thus, the grain number per panicle (209.39 grains) of D3 displayed no significant difference from that (190.18, 212.07, and 223.29 grains) of S2 (*qGPP6*), S3 (without GPP QTL), and Xihui18. This suggests that the segment on chromosome 7 without the GPP locus has no significant effect on the expression of *qGPP6* (**Figure 4D**).

The interaction of *qRLW4* (*a* = −0.13) and *qRLW7-2* (*a* = −0.01) produced an epistatic effect of 0.02, resulting in −0.12 of the genetic effect for the rate of length to width in D1, parallel to the additive effect of *qRLW4*. Accordingly, the rate of length to width (3.18) of D1 showed no significant difference from that (3.15) of S1 containing *qRLW4* while significantly lower than that (3.41 and 3.39) of Xihui18 and S3 (*qRLW7-2*). The interaction of *qRLW4* (*a* = −0.13) and *qRLW6* (*a* = −0.06) produced an epistatic effect of 0.15, causing −0.04 of the genetic effect of D2, larger than the additive effects of each single QTL. Accordingly, the rate of length to width (3.35) of D2 was significantly higher than that (3.15 and 3.29) of S1 (*qRLW4*) and S2 (*qRLW6*) while significantly lower than that (3.41) of Xihui18. However, *qRLW6* (*a* = −0.06) and *qRLW7-2* (a = −0.01) belonged to independent inheritance in D3. Thus, the genetic effect of D3 was −0.07 for the rate of length to width. Accordingly, the rate of length to width of D3 (3.14) was significantly lower than that (3.29, 3.18, and 3.41) of S2 (*qRLW6*), S3 (*qRLW7-2*), and Xihui18 (**Figure 4E**).

The interaction of *qGWT4* (*a* = −0.35 g) and *qGWT7* (*a* = −0.29 g) in D1 produced an epistatic effect of −1.32, resulting in −1.96 g of the genetic effect of D1, smaller than any of the additive effect of each single QTL. Thus, the 1000-grain weight of D1 (25.23 g) was significantly lighter than that (28.43, 28.56, and 29.13 g) of S1 (*qGWT4*), S3 (*qGWT7*), and Xihui18. The interaction of *qGWT4* (*a* = −0.35 g) and *qGWT6* (*a* = 0.96 g) produced an epistatic effect of 0.95, resulting in 1.56 g of the genetic effect of D2, larger than any of the additive effects of each single QTL. Accordingly, the 1000-grain weight of D2 (32.24 g) was significantly heavier than that (28.43, 31.05, and 29.13 g) of S1 (*qGWT4*), S2 (*qGWT6*), and Xihui18. However, *qGWT6* (*a* = 0.96 g) and *qGWT7* (*a* = −0.29 g) were inherited independently in D3. Therefore, the genetic effect on 1000-grain weight was 0.67 g in D3, larger than the additive effect of *qGWT7* whereas less than that of *qGWT6*. Accordingly, the 1000-grain weight of D3 (30.23 g) was significantly heavier than that (29.13 g) of Xihui18 and S3 (28.56 g) containing *qGWT7* while significantly lighter than that of S2 (31.05 g) carrying *qGWT6* (**Figure 4F**).

### 3.5 Genetic analysis and fine-mapping of the major QTLs for grain length—*qGL4*

The recipient parent, Xihui18, has a long-grain phenotype, while the recipient parent, Jinhui35, has a short-grain phenotype. The grain length of Z691 is significantly shorter than that of Xihui18. An F_3_ population was further developed from a self-cross of an F_2_ recombinant plant containing only the heterozygous *qGL4* locus. In this F_3_ population of 631 individual plants, the grain lengths’ frequency distribution was bimodal, with grain lengths ranging from 9.76 to 10.91 mm. The short-grain peak is mainly between 9.76 and 10.16 mm, with a total of 151 plants, and the long-grain peak is mainly between 10.16 and 10.91 mm, with a total of 480 plants. After chi-square testing, the ratio of long-grain plants (480) to short-grain plants (151) fits a 3:1 segregation ratio (χ^2^ = 0.36 < χ^2^_(0.05,1)_ = 3.84), indicating that the short grain controlled by *qGL4* in S1 is governed by an incomplete single recessive gene (**Figure 5A**). Comparing the grain length of the 151 recessive plants with that of Xihui18, it was found that the grain length of the recessive plants is significantly shorter than that of Xihui18, further indicating that the long-grain trait is incompletely dominant over the short-grain trait (**Figure 5B**).

**Figure 6:**
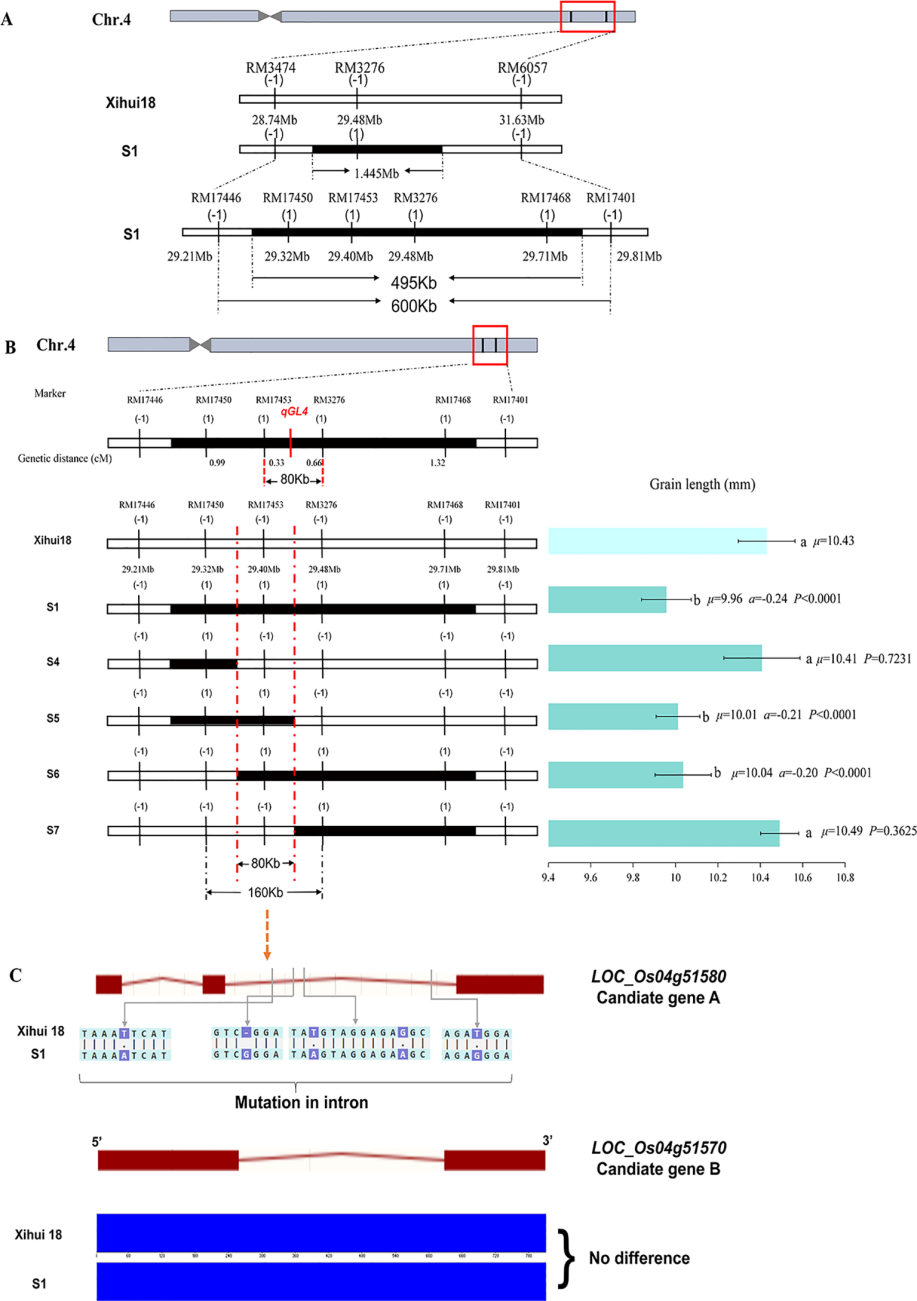
Fine-mapping and candidate gene analysis of *qGL4*. **(A)** Shorten the replacement fragment on Z691 by encryption marker. **(B)** Fine-mapping of qGL4 by linkage analysis. Diffferent small letters indicated the significant difference among the genetic materials. **(C)** DNA sequence of Candidate gene A and Candidate gene B for qGL4 in S1 compared with that of Xihui18. The green lines with arrowhead indicate the variation between the single-segment substitution lines (SSSLs) and Xihui18. The red rectangles indicate the exons, and the red lines indicate the introns. The blue rectangles indicate the sequencing peak map.

*qGL4* is a major QTL for grain length, explaining 22.14% of the variation. Genetic analysis indicates that *qGL4* from Jinhui35 is inherited as a single recessive gene in S1. Within the initial mapping interval of *qGL4*, RM3474–RM3276–RM6057, 12 new primer pairs were synthesized, of which five pairs were polymorphic, namely, RM17446, RM17450, RM17453, RM17468, and RM17401. However, the bands of RM17446 and RM17401 were consistent with the recipient Xihui18 in S1, leaving only three pairs of markers as substitution segment markers. Consequently, the estimated length of the substitution segment in S1 was shortened from the original 1.445 Mb to 495 kb. The maximum length of the substitution segment was shortened from the original 2.89 Mb to 600 kb (**Figure 6A**). Subsequently, using the dense markers on these three substitution segments and the original substitution markers, linkage analysis with *qGL4* was conducted by 151 recessive plants (short grain) in the F_3_ population. By calculating their genetic distances, *qGL4* was ultimately localized to an 80-kb interval between RM17453 and RM3276 (**Figure 6B**).

Furthermore, to construct an SSSL containing the shortest length of *qGL4*, MAS was conducted on all substitution markers for 100 plants from the F_3_ population, and a total of four SSSLs (S4–S7) were constructed for this locus. S1 (with a grain length of 9.96 mm), which contained the substitution segment RM17446–RM17450-RM17453-RM3276-RM17468–RM17401, S5 (with a grain length of 10.01 mm), which contained the substitution segment RM17446–RM17450-RM17453–RM3276, and S6 (with a grain length of 10.04 mm), which contained the substitution segment RM17450–RM17453-RM3276-RM17468–RM17401, all had grain lengths significantly shorter than those of Xihui18 (10.43 mm), with overlapping substitution regions at RM17453. However, S4, containing the substitution segment RM17446–RM17450–RM17453 with a grain length of 10.41 mm, and S7, containing the substitution segment RM17453–RM3276-RM17468–RM17401 with a grain length of 10.49 mm, showed no significant difference in grain length compared to Xihui18 (10.43 mm). According to the theory of overlapping substitution mapping, *qGL4* was located within the overlapping intervals of S1, S5, and S6. Ultimately, *qGL4* was localized to an estimated length of 80 kb within RM17450–RM17453–RM3276, with the maximum length being 160 kb (**Figure 6B**).

Within the interval, two prospective genes of *qGL4* were determined and named Candidate gene A and Candidate gene B (**Figure 6C**). The Candidate gene A (*LOC_Os04g51580*) exhibited multiple differences between Xihui18 and S1, including four single-nucleotide polymorphism (SNP) differences and one base insertion in the intron region, which may affect the alternative splicing of this gene in S1. As for the Candidate gene B (*LOC_Os04g51570*), there were no differences between Xihui18 and S1. Therefore, *LOC_Os04g51580* (Candidate gene A) can be considered the candidate gene for *qGL4*.

### 3.6 Overlapping substitution mapping for the major grain width QTL—*qGW6*

**Figure 7:**
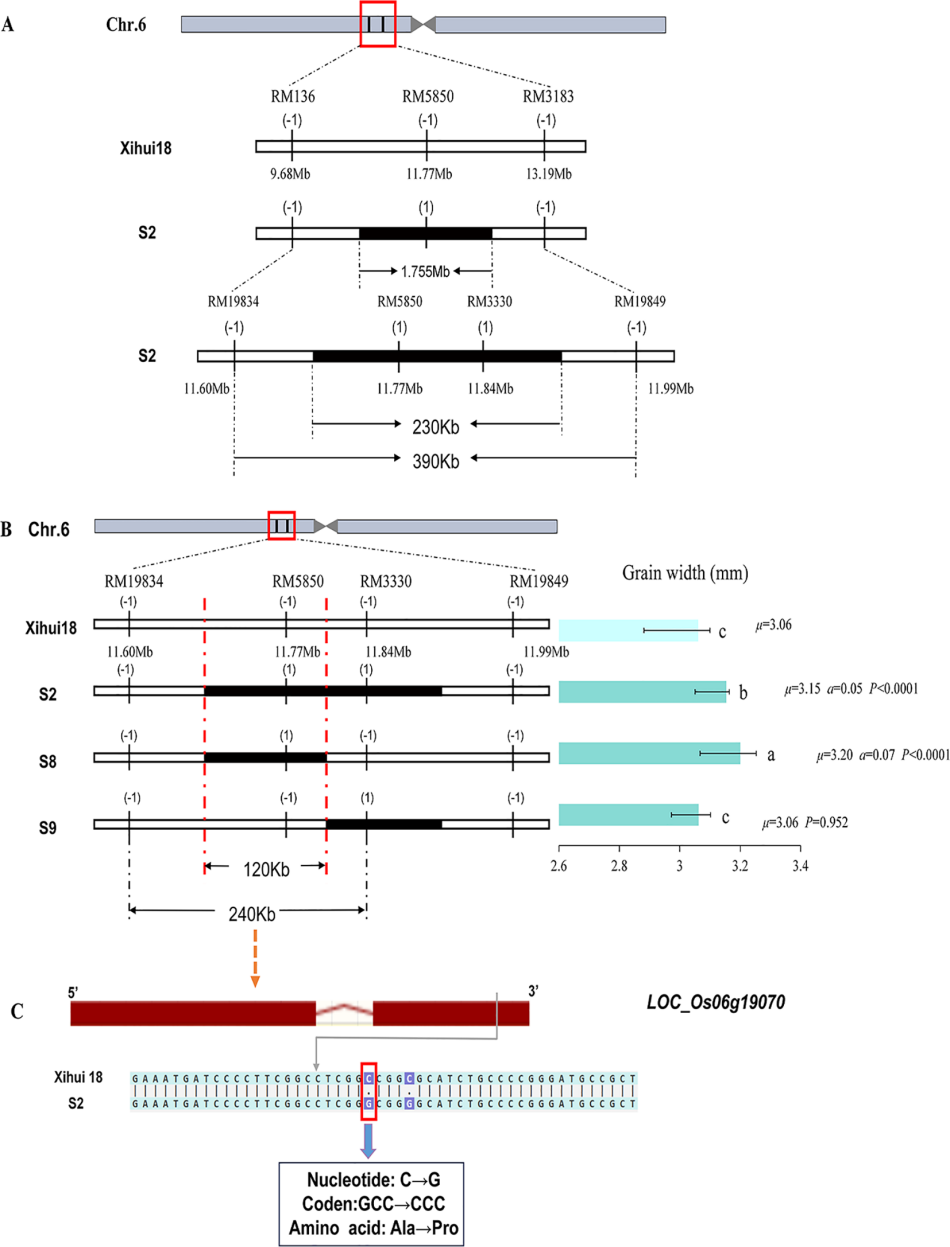
Overlapping group substitution mapping and candidate gene analysis of *qGW6*. **(A)** Shorten the replacement fragment on Z691 by encryption marker. **(B)** Fine-mapping of *qGW6* by linkage analysis. Different small letters indicated significant difference among the genetic materials. **(C)** DNA sequence of Candidate gene C for *qGW6* in S2 compared with that of Xihui18. The red rectangles indicate the exons, and the red lines indicate the introns. The green lines with arrowhead indicate the variation between the single-segment substitution lines (SSSLs) and Xihui18.

Furthermore, *qGW6* is a major QTL for grain width located in the RM136–RM5850–RM3183 interval of S2, explaining 10.61% of variation. Furthermore, we synthesized 14 new primer pairs, of which three showed polymorphisms between Xihui18 and Jinhui35, namely, RM19834, RM3330, and RM19849. However, the bands of RM19834 and RM19849 were consistent with the recipient Xihui18 in S2, leaving only one pair of markers as substitution segment markers. Consequently, the estimated length of the substitution segment in S2 was reduced from the original 1.755 Mb to 230 kb, and the maximum length of the substitution segment was shortened from the original 3.51 Mb to 390 kb (**Figure 7A**).

Using an F_2_ recombinant plant containing only the *qGW6* locus for self-crossing to develop an F_3_ population, we conducted MAS on 100 individual plants with substitution markers, and we constructed a total of two SSSLs (S8 and S9) for this locus. S2 (with a grain width of 3.15 mm), containing the substitution segment RM19834–RM5850-RM3330–RM19849 and S8 (with a grain width of 3.20 mm), containing the substitution segment RM19834–RM5850–RM3330, had significantly larger grain widths than Xihui18 (3.06 mm), and they included an overlapping substitution interval RM19834–RM5850–RM3330. Additionally, S9, containing the substitution segment RM5850-RM3330–RM19849, had a grain width (3.06 mm) similar to that (3.06 mm) of Xihui18. Applying the overlapping substitution mapping theory indicates that *qGW6* was located within the overlapping interval of S2 and S8. Ultimately, *qGW6* was localized to an estimated length of 120 kb within RM19834–RM5850–RM3330, with the maximum estimated length being 240 kb (**Figure 7B**).

Within the interval, *LOC_Os06g19070* was predicted as the candidate gene of *qGW6*. It exhibited two SNP differences at the 1594th and 1598th bases of its exon; however, only one resulted in an amino acid change (**Figure 7C**).

## 4 Discussion

### 4.1 The nine single-segment substitution lines dissected from CSSL-Z691 have significant application value in rice breeding

Most of the agronomic traits in rice are quantitative traits controlled by multiple genes, such as plant height, grain length, grain width, and yield (Ren et al., 2023; Xing and Zhang, 2010). Accurately mapping these QTLs is fundamental to studying these important agronomic traits. However, the genetic background of primary mapping populations is very complex, which is not conducive to accurate QTL mapping and identifying individual QTL genetic effects (Xu et al., 2017). CSSLs have only a few chromosome segments from the donor parent, with the rest of the genetic background consistent with the recipient parent. Therefore, using CSSLs greatly improves the efficiency of QTL detection and lays the foundation for molecular design breeding in rice (Li et al., 2022b). In this study, the dense-grain and large-grain rice CSSL Z691 was identified as containing four substitution segments from Jinhui35 based on the genetic background of Xihui18, with a total length of substitution segments of 16.26 Mb and an average length of substitution of 4.045 Mb. However, Z691 was not still an excellent breeding material because contained some unfavorable traits, such as the grain number per panicle significantly decreased by 21.18%, the grain length decreased by 18.29%, and the yield per plant decreased by 21.52% compared with those of the large panicle type restorer line Xihui18. Thus, to create more ideal materials for direct breeding and basic study in gene functional analysis, nine SSSLs and three DSSLs were further constructed carrying various QTLs by QTL mapping and MAS through Xihui18/Z691. S1 harboring *qGL4* (*a* = −0.24 mm), *qGW4* (*a* = 0.05 mm), *qGWT4* (*a* = −0.35 g), *qPL4* (*a* = −0.51 cm), and *qGPP4* (*a* = 10.32 grains) displayed 9.66 mm of grain length, 3.16 mm of grain width, 28.43 g of 1000-grain weight, 28.16 cm of panicle length, and 243.94 grains per panicle, which belonged to a multiple grain and large grain type breeding material. S2 containing *qGW6* (*a* = 0.05 mm), *qGWT6* (*a* = 0.96 g), *qPL6* (*a* = −0.88 cm), and *qGPP6* (*a* = −16.55 grains) exhibited 3.16 mm of grain width, 31.05 g of 1000-grain weight, 27.43 cm of panicle length, and 190.18 grains per panicle, which still belonged to a multiple grain and large grain type breeding material. S3 carrying *qGL7-2* (*a* = −0.06 mm), *qGWT7* (*a* = −0.29 g), and *qPL7-2* (*a* = −0.71 cm) showed 10.31 mm of grain length, 28.56 g of 1000-grain weight, 27.76 cm of panicle length, and 212.07 grains per panicle, which also belonged to a multiple and large grain type breeding material. In comparison, S1 and S2 are better breeding materials. However, in the future, S3 can be edited using the Cas9 technique to knock out *qGL7-2*, *qGWT7*, and *qPL7-2*, so S3 is potentially a great breeding material. In addition, Deng et al. (2023), using Xihui18 as the recipient parent, developed four SSSLs and detected 25 QTLs, including *qPN3*, *qGW3*, *qPN4*, and *qPL4*. Liang et al. (2021) constructed four SSSLs on the genetic background of Xihui18 and detected eight QTLs for grain size, including *qGL3-2*, *qGL3-1*, and *qGL7*. Li et al. (2022a) developed six SSSLs on the genetic background of Xihui18 and detected 11 QTLs, including *qGL11*, *qGL3*, *qGL5*, and *qGW5*. Sun et al. (2022) developed six SSSLs on the genetic background of Xihui18 and detected 15 QTLs, including *qPN5*, *qGL1*, *qGL2*, and *qGL5*. Nevertheless, these SSSLs are all different from those constructed in this study. Thus, the new SSSLs dissected from Z691 are important to perfect the SSSL library. Notably, using SSSLs as a platform to pyramid favorable alleles for breeding new varieties has become a reality (Dai et al., 2015). Some SSSLs have been successfully used to design a series of new cultivars, such as Hua Xiaohei No. 1 and Hua Biao No. 1 (Zhang, 2021). In the future, these SSSLs can be re-sequenced to ensure a completely purified background with the price of high-throughput sequencing becoming cheaper. Therefore, these SSSLs lay a solid foundation for the genetic analysis of QTLs for grain size and other traits and establish a basis for designing breeding rice varieties that meet production needs using SSSLs as the platform.

### 4.2 Comparison of the QTLs carried by Z691 with the reported genes

We utilized the secondary F_2_ population of Xihui18/Z691 and the three dissected single-segment substitution lines (S1–S3) to identify 20 QTLs for important agronomic traits in rice. By comparing with genes previously mapped or cloned in these intervals, we found that *OsHXK1* is located within the substitution intervals of *qGPP7* and *qSSR7*. *qGPP7* and *qSSR7* are linked to the same substitution interval (RM5481–RM1135–RM320), and they may represent pleiotropy. *OsAGO2* is also involved in the development of rice. Further research revealed that the knockdown of *OsAGO2* leads to a significant upregulation of the expression level of the hexokinase gene *OsHXK1* and a significant decrease in the methylation level of its promoter. That indicates that *OsAGO2* directly binds to the promoter region of *OsHXK1* and regulates the expression of *OsHXK1* through DNA methylation, thereby affecting pollen development and significantly reducing the grain number and the seed setting ratio (Zheng et al., 2019). Therefore, *OsAGO2* can be considered a candidate gene for *qGPP7* and *qSSR7*.

*qGL4*, *qRLW4*, and *qGWT4* are located within the substitution interval (RM3474–RM3276-RM5611–RM6057), where the gene *FLO2* was found. The *FLO2* mutation results in smaller grains and reduced starch quality. *FLO2* is highly expressed in developing seeds, coinciding with the starch and storage protein synthesis period, and is also highly expressed in leaves. The overexpression of *FLO2* can significantly increase grain size, and *FLO2* plays a key regulatory role in grain size and starch quality by affecting the accumulation of storage substances in the endosperm (She et al., 2010). Compared to the wild type (WT), at the seedling stage, the *DHT1* mutant exhibits slower growth and smaller roots, although the number of tillers at this stage does not significantly differ from that of the WT. However, at the heading stage, the *DHT1* mutant has a reduced plant height due to shorter internodes and has more tillers. Histological analysis indicates that the stem cells of the *DHT1* mutant are smaller than those of the wild type, with reduced cell width and length (Liu et al., 2022).

Similarly, S1 harboring *qRLW4* and *qGWT4* displayed shorter grain length and lighter grain weight than Xihui18. This suggests that *FLO2* can be a candidate gene for *qGL4*, *qRLW4*, and *qGWT4*. In addition, we further fine-mapped the *qGL4* into 160 kb of the largest substitution interval and found a candidate gene *LOC_Os04g51580* (a leucine-rich repeat-containing protein) by DNA sequencing. However, *FLO2* was not within the 160-kb interval. *OsPEX1*, encoding a leucine-rich repeat extension protein, regulates grain size and rice quality (Luan et al., 2021). As for whether *FLO2* or *LOC_Os04g51580* is the target gene of *qGL4*, it needs to be confirmed by a genetic complementary test in the future.

*qGW6*, *qRLW6*, and *qGWT6* are located within the same substitution interval (RM136–RM5850–RM3183) and may exhibit pleiotropy. Within this interval, the gene *OsGSR1* has been found to regulate cell growth rather than cell proliferation by positively regulating the gibberellin signaling pathway by modulating the response and biosynthesis of gibberellin, thereby controlling rice grain size. The knockout of *OsGSR1* results in reduced grain size and weight and a decrease in gibberellin content in the young panicle, while the overexpression of *OsGSR1* leads to an increase in grain size and weight (Shi et al., 2020). Additionally, gene expression analysis has shown that *OsGSR1* is also regulated by brassinolide treatment and accumulates in the panicle, indicating that *OsGSR1* may also be involved in the brassinosteroid-regulated pathway for controlling grain length (Tang et al., 2020). S2 containing *qGW6*, *qRLW6*, and *qGWT6* from Huhan3 exhibited an increase in grain width and weight compared with Xihui18. This suggests that *OsGSR1* can be a candidate gene for *qGW6*, *qRLW6*, and *qGWT6*.

Furthermore, *qGW6* was limited to 240 kb of the largest substitution interval through overlapping substitution mapping, *LOC_Os06g19070* was determined as the candidate gene by DNA sequencing, and *OsGSR1* was not within the 240-kb interval. *LOC_Os06g19070*, also a cytochrome P450 protein, controls grain width by regulating the gibberellin (GA) content in rice spikelets (Dang et al., 2024). *GNS4* also encodes cytochrome P450 protein by the brassinosteroid (BR) signaling pathway to regulate rice grain size. The promoter variation of the gene in the *gns4* mutant exhibits a small grain size (Zhou et al., 2017). A genetic complementary test must also verify whether *LOC_Os06g19070* or *OsGSR1* is the candidate gene for *qGW6*.

*qGW7*, *qGL7-2*, and *qGWT7* are linked to the marker RM1279 (19.67 Mb), and near this marker, *OsBZR1* is a downstream signaling molecule in BR signaling. Compared to the non-transgenic control, the overexpression of *OsBZR1* leads to an increase in sugar accumulation in developing anthers and seeds, as well as an increase in grain yield, with enhancements in grain length, width, thickness, 1000-grain weight, and the spikelet number per panicle. In contrast, suppressing *OsBZR1* expression results in pollen maturation defects, reduced seed size and weight, and decreased starch accumulation. Furthermore, during the development of rice pollen and seeds, *OsBZR1* can directly promote the expression of CSA, which triggers the expression of genes related to sugar allocation and metabolism (Zhu et al., 2015). S3 carrying *qGL7-2* and *qGWT7* from Jinhui35 displayed a decrease in grain length and grain weight. Therefore, *OsBZR1* may be a candidate gene for *qGW7*.

*qGPP4* is linked to the marker RM3276 (29.48 Mb), and *LVPA4* and *OsNPF7.4* are very close to *qGPP4* in position. Compared to the recurrent parent, the near-isogenic line NIL-LVPA4^LT^ showed an increase of 7.8% in the total area of the large vascular bundles in the neck of the panicle, an increase of 12.7% in the phloem of the large vascular bundles, and an increase of 9.4% in the number of large vascular bundles. Still, there were no significant differences in the number of small vascular bundles and the area of the xylem of the large vascular bundles. In addition, the size of the panicle; the length, width, and area of the flag leaf; and the leaf area index significantly increased, especially with a 17.2% increase in the spikelet number per panicle, a 19.7% increase in the number of secondary branches, a 22.5% increase in grain number per panicle, and an increase in the content of non-structural carbohydrates in the sheath of the stem at the heading stage, ultimately leading to a 7.6%–9.6% increase in the yield of NIL-LVPA4^LT^ in plot trials while also improving rice quality. The study shows that the single gene *LVPA4* improves rice yield and quality through the coordinated action of source, sink, and flow (Zhai et al., 2022). The overexpression of *OsNPF7.1* or the knockout of *OsNPF7.4* can increase seedling biomass, the number of tillers, grain number, and yield per plant; the phenotype is opposite when RNAi silencing *OsNPF7.1* or overexpressing *OsNPF7.4* (Huang et al., 2019). S1 harboring *qGPP4* showed an increase in grain number per panicle (243.94 grains) compared to Xihui18. Therefore, based on the gene function and location, *LVPA4* and *OsNPF7.4* may be candidate genes for *qGPP4*.

The remaining seven QTLs (*qPL7*, *qNPB7*, *qGL7-1*, *qRLW7*, *qPL4*, *qPL6*, and *qGPP6*) have yet to be reported. Therefore, they may be newly identified QTLs. The results of this study establish a solid foundation for further functional analysis of these QTLs and “breeding by design.”

### 4.3 Understanding the epistasis effects among QTLs can significantly impact genotype–phenotype prediction and is crucial for molecular design breeding in the future

The inheritance of most crop agronomic traits is highly complex and influenced by the environment. The importance of epistasis as the genetic basis of complex traits has been confirmed on the foundation of classical quantitative genetic studies. Experiments on the changes in allele frequency of isoenzyme markers have also revealed that epistasis between multiple loci may play an important role in the evolution of complex traits in plant species (Allard, 1996; Zhuang et al., 2002). We constructed three SSSLs and three DSSLs for the target QTLs in the progeny of Xihui18/Z691. We analyzed the epistasis effects of QTLs related to six important rice agronomic traits. The results showed that even the same QTL interacting with different QTLs produces different genetic models, displaying independent inheritance or various epistatic effects. For instance, the interaction of *qGW4* (*a* = 0.05 mm) and *qGW6* in D2 (*a* = 0.05 mm) resulted in an epistatic effect of −0.03, whose genetic effect was 0.07 mm for grain width, greater than any additive effect of single QTL, which resulted in that the grain width (3.20 mm) of D2 being significantly higher than that of Xihui18, S3 containing *qGW4*, and S4 containing *qGW6*. That is to say, if the wide grain is a breeding goal, we can select the two QTL pyramiding. The interaction of *qGWT4* (*a* = −0.35 g) and *qGWT6* (*a* = 0.96 g) in D2 produced an epistatic effect of 0.95, which increased the 1000-grain weight genetically by 1.56 g, larger than the additive effect of any single QTL. If large grain is our breeding goal, *qGWT4* and *qGWT6* can be selected for breeding by design, which can increase 1000-grain weight by 1.56 g predictably. As per our prediction, the 1000-grain weight of D2 (32.24 g) was significantly heavier than that of S1 (*qGWT4*), S2 (*qGWT6*), and Xihui18. However, *qGWT6* (*a* = 0.96 g) and *qGWT7* (*a* = −0.29 g) inherited independently in D3. The genetic effect on 1000-grain weight in D3 was 0.67 g. Thus, due to the different genetic models among QTLs, it is essential to identify each QTL and its genetic models by SSSLs for breeding by design.

As each SSSL carries more than one QTL for different traits, it is necessary to evaluate the breeding value of DSSLs or SSSLs comprehensively. In D1, the genetic effect for grain length after QTL pyramiding was −0.51 mm (less than the additive effects of *qGL4* and *qGL7-2*), that for grain width was −0.03 mm (smaller than the additive effect of *qGW4*), that for 1000-grain weight was −1.96 g (less than the additive effects of *qGWT4* and *qGWT7*), that for panicle length was −2.21 cm (less than the additive effects of *qPL4* and *qPL7-2*), and that for grain number per panicle was equal to the additive effect (10.32 grains) of *qGPP4*. Thus, after pyramiding these QTLs, the dual-segment substitution line (D1) displays the phenotype of multiple grains (225.23 grains) and smaller grains (25.23 g) compared to Xihui18. According to Calingacion et al. (2014), grain shapes larger than 3.1 in length to width belong to the slender type. Thus, D1 still exhibits a slender type (3.18 for length to width), although less than 3.41 for the rate of length to width of Xihui18. In D2, the genetic effect for grain number per panicle after QTL pyramiding was −19.43 grains (less than the additive effect of *qGPP4*, while equal to that of *qGPP6*), that for panicle length was −0.51 cm (equal to the additive effects of *qPL4* and *qPL6*), that for grain length was 0.10 mm (larger than the additive of *qGL4*), that for grain width was 0.07 mm (larger than the additive effects of *qGW4* and *qGW6*), and that for 1000-grain weight was 0.95 g (equal to the additive effect of *qGWT6*, while larger than that of *qGWT4*). Thus, after the pyramiding of these QTLs, the phenotype of D2 displayed less grain number (171.97 grains per panicle) and large grains (32.24 g) compared to Xihui18, a large panicle type *indica* restorer line. The grain type still belongs to the slender type (3.25 for the rate of length to width) compared to that (3.41) of Xihui18. In D3, the genetic effect for grain length after QTL pyramiding was equal to the additive effect (−0.06 mm) of *qGL7-2*, that for grain width was 0.12 mm (larger than the additive effect of *qGW6*), that for 1000-grain weight was 0.67 g (larger than the additive of *qGWT7* while smaller than that of *qGWT6*), that for panicle length was 0.51 cm (larger than the additive effects of *qPL6* and *qPL7-2*), and that for grain number per panicle was −1.34 grains (parallel to the additive effect of *qGPP6*). Thus, after pyramiding these QTLs, D3 displayed large grain type (30.23 g for 1000-grain weight) and large panicle type (30.20 cm) with 209.39 grains per panicle compared to Xihui18. The grain shape (3.14 for the rate of length to width) still belongs to slender, while it becomes short-wider compared with that (3.41) of Xihui18. Altogether, after pyramiding different QTLs, D1, D2, and D3 displayed various phenotypes for increasing yield, and all can be potentially useful breeding materials. Similarly, Xu et al. (2023) produced higher yield dual-segment substitution lines (D2 and D3) and triple-segment substitution lines (TSSLs; T1 and T2) by pyramiding various QTLs compared to Xihui18. All these SSSLs, DSSLs, and TSSLs will benefit the breeding by design on the platform of SSSL-Xihui18 in the future.

## 5 Conclusion

We dissected three rice SSSLs and DSSLs on the genetic background of Xihui18 from the progeny of Xihui18/Z691. We found 15 QTLs associated with yield-related traits. S1 harboring *qGL4* (*a* = −0.24 mm), *qGW4* (*a* = 0.05 mm), *qGWT4* (*a* = −0.35 g), *qPL4* (*a* = −0.51 cm), and *qGPP4* (*a* = 10.32 grains) displayed multiple and large grains, which are a good high-yield breeding material. Owing to various genetic models after pyramiding different QTLs, it is essential to identify the additive effect of each single QTL and the epistatic effect between QTLs using SSSLs and DSSLs in future breeding by design. D1, D2, and D3 displayed various phenotypes for increasing yield, and all can be potentially useful breeding materials. Finally, our fine-mapping and candidate gene determination of *qGL4* and *qGW6* lays a good foundation for map-based cloning and functional studies of these genes, which benefits future breeding by design.

## Data Availability

The original contributions presented in the study are included in the article/**Supplementary Material**. Further inquiries can be directed to the corresponding author.

## Supplementary material

Supplementary Figure 1 The flow chart of Z691 development.
