# Supplementary figures and images for: Genetic dissection of QTL for important agronomic traits and fine-mapping of qGL4 and qGW6 based on a short-width grain rice CSSL-Z691
Source: Front Plant Sci. 2025 Mar 10;16:1539625. doi: 10.3389/fpls.2025.1539625 (PMC11931059; doi:10.3389/fpls.2025.1539625)

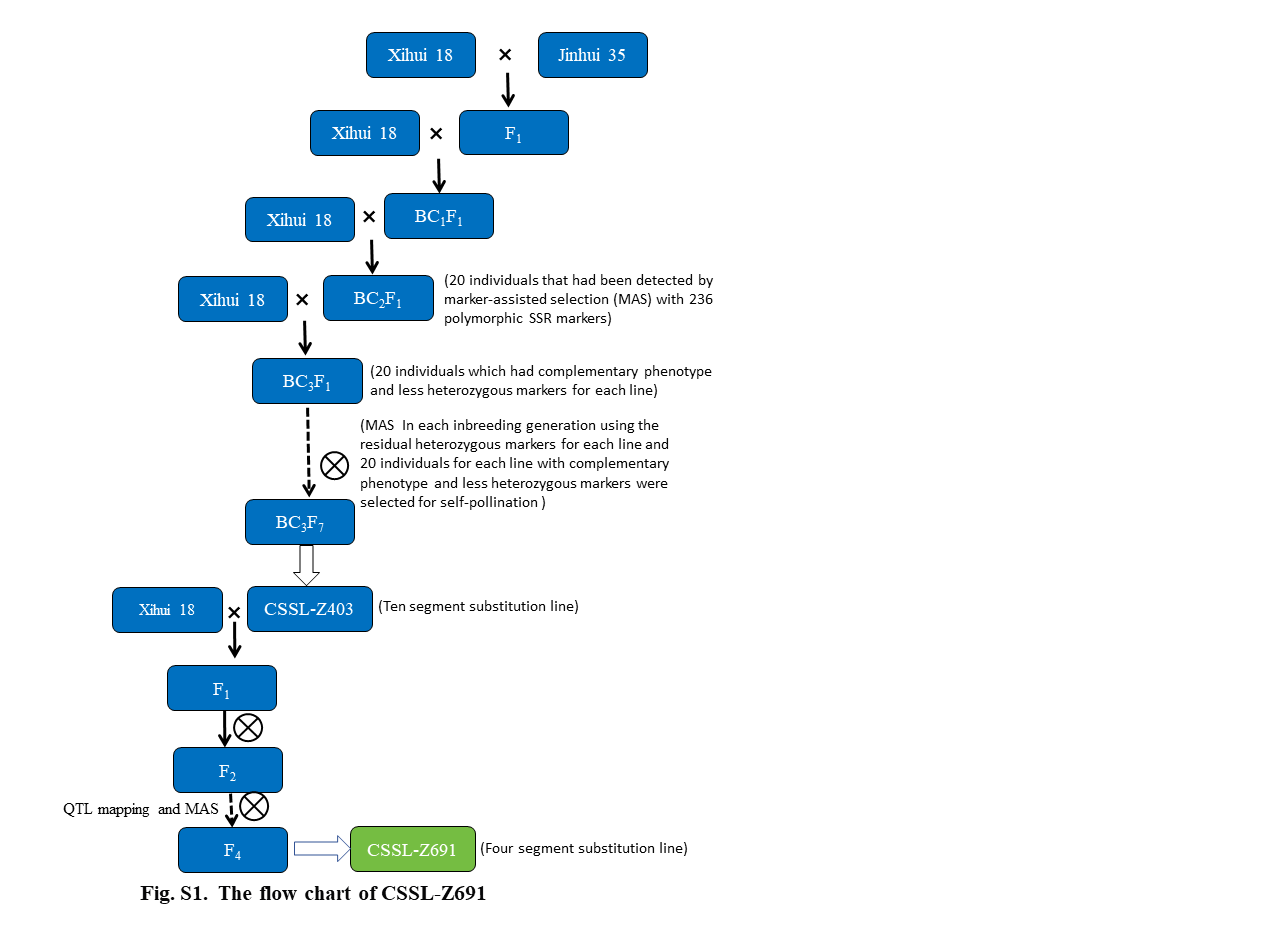

Supplement: Supplementary Figure 1 — The flow chart of Z691 development. [file Image1.tif]
